# Supplementary material for: A holistic view of the dynamics of long-lived valley polarized dark excitonic states in monolayer WS2
Source: Nat Commun. 2025 Jul 10;16:6385. doi: 10.1038/s41467-025-61677-2 (PMC12246435; doi:10.1038/s41467-025-61677-2)
Supplement: Supplementary file 1 — Supplementary Information [file 41467_2025_61677_MOESM1_ESM.pdf]

Supplementary Information

1. TR-XUV-ARPES Energy resolution
2. Valence band linewidth of the WS_2 monolayer sample
3. Extraction of electron populations in the spin-split bands
4. Equalization of the photoemission matrix elements
5. Symmetrization of 2D ARPES data using a 120° rotating average
6. Electron and hole density
7. Rate equations
8. Spin dark excitons populations and degree of polarization
9. Dispersion of the exciton electrons
10. Intensity dependent bright exciton momentum distribution and valley polarization

1. TR-XUV-ARPES Energy resolution

We determined our instrument energy resolution by measuring the Fermi edge of an Au(111) sample at 100 K using the 21.7 eV probe. As seen in Fig.S1, we find a 12-88% width of ~50 meV. Additionally, we performed the same measurement using directly the 515nm pump with a known spectral width of 12 meV (measured with an optical spectrometer) for which we extract a 12-88% width energy resolution of 42 meV. By deconvolution, we extract a contribution of 40 meV originating from the instrument, temperature and sample. Using this, we find the XUV probe contribution to be of 30 meV.

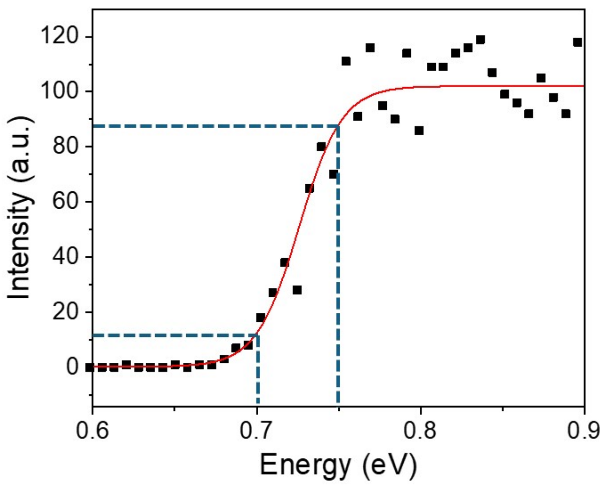

Fig.S1: Experimental energy resolution. Energy distribution curve of Au(111) around the Fermi edge at 100 K with the 21.7 eV probe.

2. Valence band linewidth of the WS_2 monolayer sample

In the sample studied in the manuscript and corresponding ARPES data, we find a top valence band (VB1) FWHM linewidth of 88meV without optical pump obtained from a fit with a Gaussian function (see Fig.S2a). At the instant of photoexcitation with an intensity corresponding to an exciton density of 4×10^{11} cm^{-2}, we find minimal effect from the pump with a measured top valence band linewidth of 100meV.

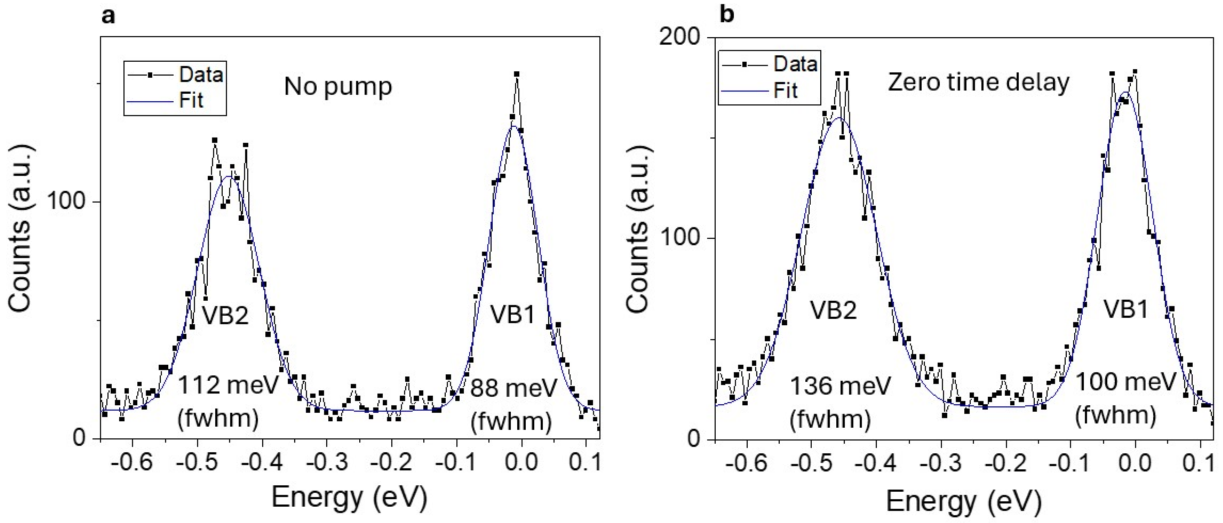

Fig.S2: Valence bands linewidth. Energy distribution curves for (a) no optical excitation showing a FWHM linewidth of 88meV for the top valence band (VB1) and 112meV for the lower valence band (VB2) and (b) at zero-time delay showing minimal effect of pump induced broadening in VB1 and VB2.

3. Extraction of electron populations in the spin-split bands

To acquire the electron population of the spin-split states, we extract energy distribution curves (EDC), as in Fig.S3, obtained by integrating a 0.1 x 0.1-Å^{-1} k-space area centered at the K and K' valleys for each time delay. For all EDC, the zero-energy reference is set to the top valence band. First, we fit the energy distribution with a single Gaussian function at the instant of photoexcitation for which the only contribution comes from the bright exciton with electrons located in the upper spin-split state. This sets the upper energy state to 2.090eV±2meV with a FWHM linewidth of 94±3meV. Then, we repeat this fitting procedure at a later time delay (3ps) once the electron population has relaxed to the lower spin-split state. We find the lower state energy at 2.051eV±3meV with a FWHM linewidth of 98± 5meV, as narrow as the linewidth measured for the upper state at zero-time delay. From 1ps to 10ps, single Gaussian functions provide excellent fit, showing that we only have contribution from the lower spin-split state. From 0.1 to 0.9 ps, the signal is a contribution from both states. Within this time window, we used a double Gaussian function for which the center energies and linewidth were fixed according to the values extracted for the upper and lower states at 0ps and 3 ps, respectively.

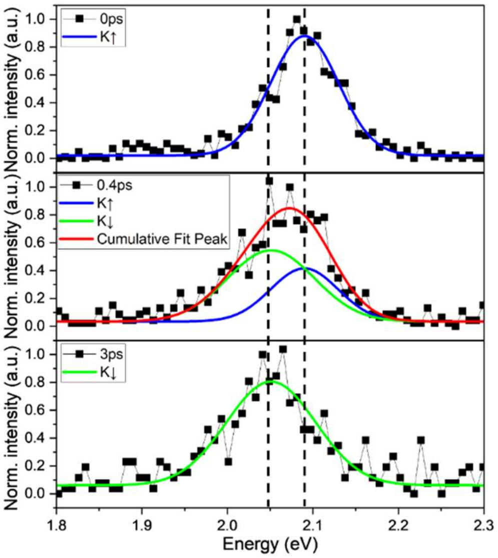

Fig.S3: Spin-split states fitting. (top panel) Energy distribution curve (black dots) and single Gaussian fit (blue) at zero-time delay, (middle panel) Energy distribution curve at 0.4 ps showing the contribution from the upper and lower spin split states fitted by a double Gaussian function and (bottom panel) Energy distribution curve at 3ps and single Gaussian fit (green).

4. Equalization of the photoemission matrix elements

The photoemission matrix elements, due to the oblique incidence angle of the XUV probe, are not uniform for a given energy across the 1st BZ [1] (see Fig. S4a). However, comparing accurately the electron populations between two adjacent K and K' valleys after valley selective photoexcitation requires their matrix elements to be equal, namely that the same electron counts are measured at the K and K' valleys when they are equally populated. To ensure that at least two K and K' valleys fulfill this criterion, we rotated the sample until the exciton-bound electron signals were equal when equally populated (see Fig.S4a-d). To verify the validity of the procedure, we measured the electron dynamics in the K and K' valleys under a linearly polarized photoexcitation (see Fig.S4e). We see that the electron counts (normalized by the total photoelectron counts to account for variation of the XUV probe intensity) in each valley are equal at each time delay, thus allowing for a quantitative analysis of the population distribution.

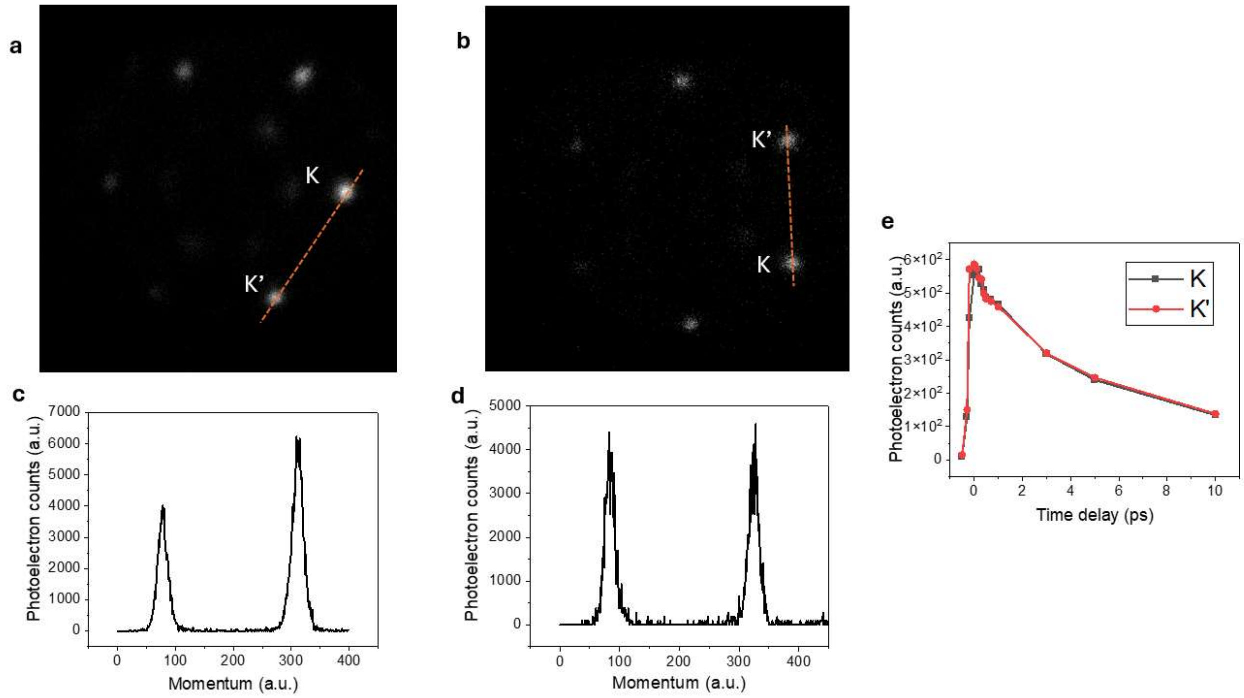

Fig.S4: Equalization of photoemission matrix elements. 2D ARPES data at the A exciton energy and at zero time delay (a) before sample rotation and (b) after a 10° rotation. (c) Photoemission intensity along a K-K' cut (orange dashed line in A) before rotation and (d) after rotation showing the equalization of intensity in the K and K' valleys under a linearly polarized photoexcitation. (e) Corresponding dynamics of the photoelectrons in the K and K' valleys showing intensity equalization at all time delays.

5. Symmetrization of 2D ARPES data using a 120° rotating average

The 2D ARPES data presented in Fig.1c, and Fig.4a,4c of the main text were plotted after performing a 120° step rotating average centered at the Γ point. This procedure symmetrizes the non-uniform photoemission intensity, as seen in Fig.S5a, originating from the variation of photoemission matrix elements due to the grazing incidence of the XUV probe. The result of the symmetrization is illustrated in Fig.S5b, showing the energy integrated signal at the energy of the exciton-bound electrons at the instant of valley selective photoexcitation (data presented in Fig.1c of the manuscript). We note that all the quantitative analysis performed in the manuscript including the energy distribution curves, momentum distribution of electrons and population and fitting of rate equations were performed with data without symmetrization. Nevertheless, we checked the validity of this operation by verifying that the extracted hole densities are the same in both cases. At the instant of photoexcitation and for the low intensity and low temperature data, we find a hole density of 4.9×10^{11} cm^{-2}, in agreement with the 4×10^{11} cm^{-2} density extracted from the raw data. The data of the holes presented in Fig.2 were also plotted using this rotating average but, in this case, centered at the K or K' valley.

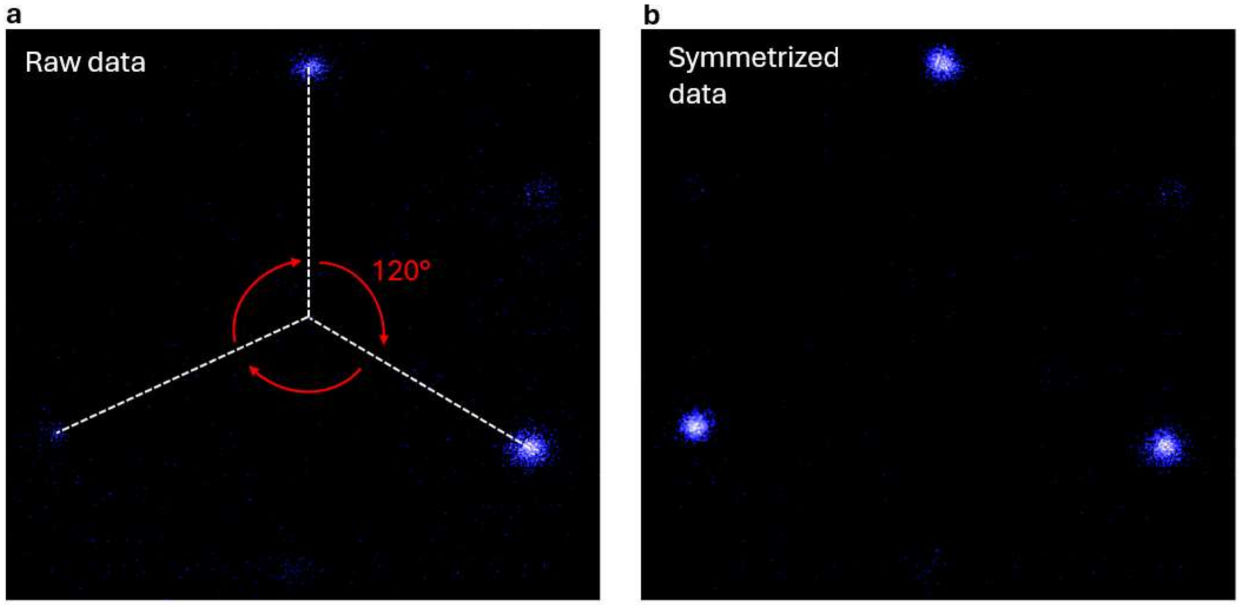

Fig.S5: Symmetrization of 2D ARPES data. 2D ARPES data at the instant of the photoexcitation (a) before performing the symmetrization by a 120° rotating average and (b) after symmetrization, clearly showing that only K valleys are populated upon valley-selective photoexcitation.

6. Electron and hole density

In Fig.S6a, we illustrate the presence of holes in the K valley at 0 ps by performing a substraction between unpumped and with pump ARPES data. In Fig.S6b, we show the corresponding momentum-integrated energy distribution curve.

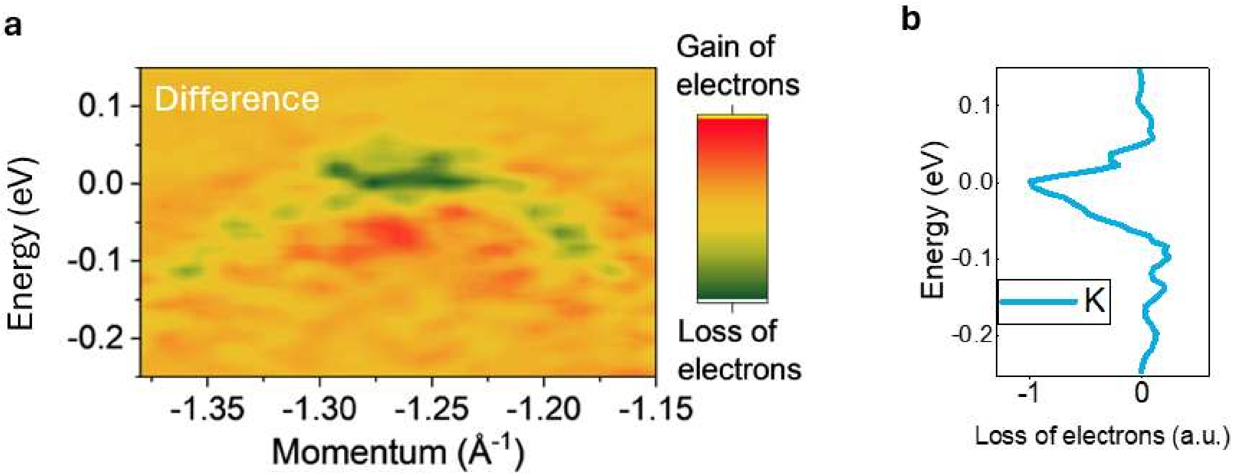

Fig. S6: Hole density. (a) ARPES data showing the difference between unpumped and pumped data at 0.1 ps displaying the hole distribution at the top valence band. (b) Corresponding energy distribution curve integrated over a 0.1×0.1-Å^{-1} k-space area showing the loss of electrons. The displayed data were obtained for a fluence of 12 µJ/cm² corresponding to a density of 4×10^{11} cm^{-2}.

We used a 0.1×0.1 A^{-1} area centered at the K and K' valleys. To obtain the density we have integrated over the same energy width the electron signals and valence band signals, i.e. 400 meV. In the Fig.S7a, we illustrate this for zero-time delay by displaying in the bandstructure the momentum-energy range used for our analysis. We also provide the associated spectra for the electrons in the K and K' valleys (see Fig.S7b). In the panel c and d, we show the spectra (here normalized with respect to the lower energy spin-split valence band) comparing unpumped and pumped data showing at zero-time delay the depletion of counts in the upper valence in the K valley and a weak change in the K' valley. The corresponding hole depletion corresponds to a total loss of 2695 counts or a fraction of 6% in the K valley (414 counts or 0.9% fraction in the K' valley) of the total density (by taking the ratio with unpumped data). From here, the density can be calculated by multiplying the fraction with the constant 2D density of states for the momentum area used, as previously shown [2], resulting in a density of 4.5×10^{11} cm^{-2} in the K valley and 7×10^{10} cm^{-2} in the K' valley.

Taking the difference between unpumped and with pump data allows to eliminate the contribution from the photoemission matrix elements and to extract the absolute hole density. To obtain the electron densities, we assume that the photoemission matrix elements do not vary with time delay

and we equalize the matrix elements between two adjacent valleys (See SI 4). Then, we equalize the electron density to the previously extracted hole density using a linear polarization excitation, for which valley have a symmetric distribution (see SI 4), and during photoexcitation. This allows us to account for the energy dependent variation of matrix element, assuming no variation within the electron signal energy linewidth.

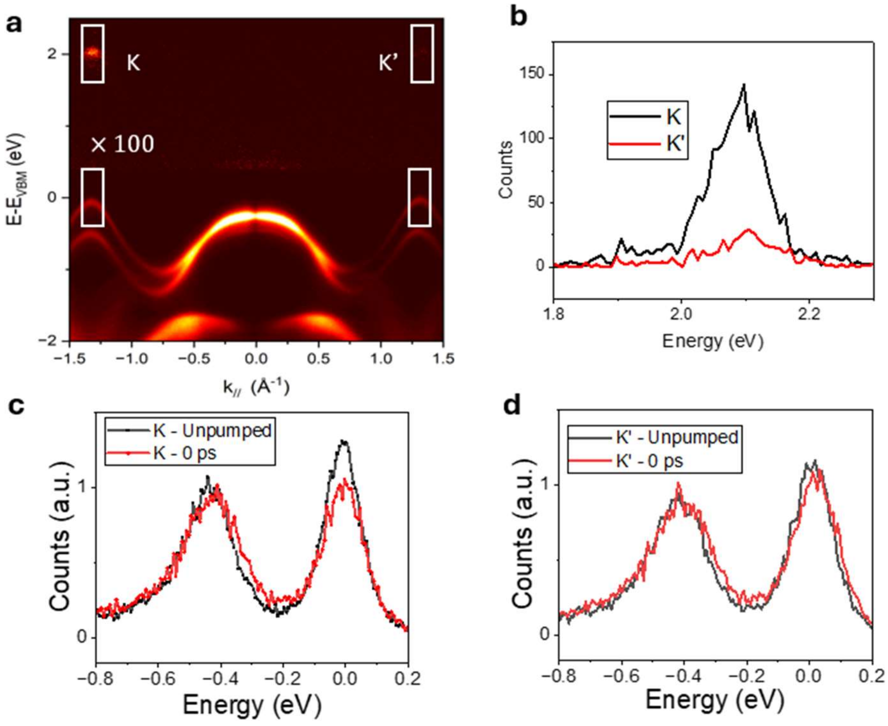

Fig.S7: Energy distribution curves. (a) Experimental bandstructure showing the energy-momentum window used for the data analysis (white rectangles). The signal above 0.4 eV have been multiplied by 100 for clarity. (b) Electron signal at 0 ps showing the contrast between K and K' valley bright excitons. (c, d) Energy distribution curves of the valence band showing majority depletion of holes in the K valley compared to the K' valley.

7. Rate equations

We can extract from our ARPES data the electron population in the spin-split states and hole population in the valence band in the K and K' valleys. Here, the contribution from the Q valleys is neglected since no measurable contribution was experimentally observed. To extract the excitonic states associated to the measured signals, we used a set of rate equations describing the temporal evolution of the population of each excitonic states of the form, as described in the manuscript:

$$\frac{dn_X}{dt} = g(t) - \sum \frac{1}{\tau_i} n_X + \sum_{i,j} \frac{1}{\tau_i} n_{X_j} \qquad (1)$$

Where τ_i includes scattering processes to other excitonic states and recombination and X is an excitonic state labeled as, for example $K_\uparrow - K_\uparrow, K_\downarrow - K_\uparrow$, etc..., where the left term corresponds to the electron location, the right term corresponds to the hole location and arrows indicate the associated spin. Experimentally, we measure the electron populations denoted $n^e_{K_\uparrow}$, $n^e_{K_\downarrow}$, $n^e_{K'_\uparrow}$, $n^e_{K'_\downarrow}$ and hole populations denoted n^h_K, $n^h_{K'}$. To access the population of each excitonic state, we fit the experimental dynamics of electron and hole populations using the rate equation model of the excitonic states (described in the next paragraph) with the constraint that excitons are composed of specific electron and hole populations. We used the transformation below that converts the constituent electron and holes into the basis of the excitonic states:

$$\begin{bmatrix} n^e_{K_\uparrow} \\ n^e_{K_\downarrow} \\ n^e_{K'_\uparrow} \\ n^e_{K'_\downarrow} \\ n^h_K \\ n^h_{K'} \end{bmatrix} = \begin{bmatrix} 1 & 0 & 0 & 0 & 1 & 0 & 0 & 0 \\ 0 & 1 & 0 & 0 & 0 & 0 & 1 & 0 \\ 0 & 0 & 1 & 0 & 0 & 1 & 0 & 0 \\ 0 & 0 & 0 & 1 & 0 & 0 & 0 & 1 \\ 1 & 1 & 0 & 0 & 0 & 1 & 0 & 1 \\ 0 & 0 & 1 & 1 & 1 & 0 & 1 & 1 \end{bmatrix} \begin{bmatrix} n_{K_\uparrow - K_\uparrow} \\ n_{K_\downarrow - K_\uparrow} \\ n_{K'_\downarrow - K'_\downarrow} \\ n_{K'_\uparrow - K'_\downarrow} \\ n_{K_\uparrow - K'_\downarrow} \\ n_{K'_\downarrow - K_\uparrow} \\ n_{K_\downarrow - K'_\downarrow} \\ n_{K'_\uparrow - K_\uparrow} \end{bmatrix} \qquad (2)$$

Initially, we solve the rate equations including all the possible scattering paths and finally discard the terms that do not contribute to the fit. In the case described in Fig.3 of the manuscript, the set of parameters contributing to the dynamics and fitting the data with high confidence are the exchange scattering τ_{ex}, the intravalley relaxation to lower spin-split state τ_{intra}, the intervalley phonon scattering of the constituent electron τ_{ph}, the intravalley scattering to the upper spin-split state $\tau_{intra,\uparrow}$ and the intervalley scattering to upper spin-split state $\tau_{ph,\uparrow}$. Since prior work established that dark states act as a reservoir slowly backscattering to bright states (included in our fit), explaining the ns-long radiative lifetime in time-resolved photoluminescence experiments[3] ,a recombination term τ_{rec} is only considered for bright excitons within our 10 ps time window. Intervalley scattering of constituent holes was found to be negligible. The generation term g(t) uses the experimental temporal resolution (150 fs Gaussian pulse). All the fitting parameters are listed in Table S1. The extracted parameters are in good agreement with previous reports discussing exchange [4] , recombination [3] and intravalley relaxation[5] .

a- Model for low intensity and low temperature

Bright excitons:

$$\frac{dn_{K_\uparrow-K_\uparrow}}{dt}=g(t)-\left(\frac{1}{\tau_{ex}}+\frac{1}{\tau_{intra}}+\frac{1}{\tau_{ph}}+\frac{1}{\tau_{rec}}\right)n_{K_\uparrow-K_\uparrow}+\frac{1}{\tau_{ex}}n_{K'_\downarrow-K'_\downarrow}+\frac{1}{\tau_{intra,\uparrow}}n_{K_\downarrow-K_\uparrow}+\frac{1}{\tau_{ph,\uparrow}}n_{K'_\uparrow-K_\downarrow} \quad (3)$$

$$\frac{dn_{K'_\downarrow-K'_\downarrow}}{dt}=-\left(\frac{1}{\tau_{ex}}+\frac{1}{\tau_{intra}}+\frac{1}{\tau_{ph}}+\frac{1}{\tau_{rec}}\right)n_{K'_\downarrow-K'_\downarrow}+\frac{1}{\tau_{ex}}n_{K_\uparrow-K_\uparrow}+\frac{1}{\tau_{intra,\uparrow}}n_{K'_\uparrow-K'_\downarrow}+\frac{1}{\tau_{ph,\uparrow}}n_{K_\downarrow-K'_\downarrow} \quad (4)$$

Spin-dark excitons:

$$\frac{dn_{K_\downarrow-K_\uparrow}}{dt}=-\left(\frac{1}{\tau_{intra,\uparrow}}+\frac{1}{\tau_{ph,\uparrow}}\right)n_{K_\downarrow-K_\uparrow}+\frac{1}{\tau_{intra}}n_{K_\uparrow-K_\uparrow}+\frac{1}{\tau_{ph}}n_{K'_\downarrow-K_\uparrow} \quad (5)$$

$$\frac{dn_{K'_\uparrow-K'_\downarrow}}{dt}=-\left(\frac{1}{\tau_{intra,\uparrow}}+\frac{1}{\tau_{ph,\uparrow}}\right)n_{K'_\uparrow-K'_\downarrow}+\frac{1}{\tau_{intra}}n_{K'_\downarrow-K'_\downarrow}+\frac{1}{\tau_{ph}}n_{K_\uparrow-K'_\downarrow} \quad (6)$$

Spin-unlike momentum-dark excitons:

$$\frac{dn_{K_\uparrow-K'_\downarrow}}{dt}=-\left(\frac{1}{\tau_{intra}}+\frac{1}{\tau_{ph}}\right)n_{K_\uparrow-K'_\downarrow}+\frac{1}{\tau_{intra,\uparrow}}n_{K_\downarrow-K'_\downarrow} \quad (7)$$

$$\frac{dn_{K'_\downarrow-K_\uparrow}}{dt}=-\left(\frac{1}{\tau_{intra}}+\frac{1}{\tau_{ph}}\right)n_{K'_\downarrow-K_\uparrow}+\frac{1}{\tau_{intra,\uparrow}}n_{K'_\uparrow-K_\uparrow} \quad (8)$$

Spin-like momentum-dark excitons:

$$\frac{dn_{K_\downarrow-K'_\downarrow}}{dt}=-\left(\frac{1}{\tau_{intra,\uparrow}}+\frac{1}{\tau_{ph,\uparrow}}\right)n_{K_\downarrow-K'_\downarrow}+\frac{1}{\tau_{intra}}n_{K_\uparrow-K'_\downarrow}+\frac{1}{\tau_{ph}}n_{K'_\downarrow-K'_\downarrow} \quad (9)$$

$$\frac{dn_{K'_\uparrow-K_\uparrow}}{dt}=-\left(\frac{1}{\tau_{intra,\uparrow}}+\frac{1}{\tau_{ph,\uparrow}}\right)n_{K'_\uparrow-K_\uparrow}+\frac{1}{\tau_{intra}}n_{K'_\downarrow-K_\uparrow}+\frac{1}{\tau_{ph}}n_{K_\uparrow-K_\uparrow} \quad (10)$$

Fitted quantity	Fitting parameter	Value (ps)
Exchange scattering	τ_{ex}	$0.35^{+0.08}_{-0.12}$
Intravalley relaxation to lower spin split state	τ_{intra}	$4^{+1.4}_{-1.1}$
Intervalley phonon scattering	τ_{ph}	$0.9^{+0.35}_{-0.21}$
Radiative recombination	τ_{rec}	$0.35^{+0.27}_{-0.12}$
Intravalley scattering to upper spin split state	$\tau_{intra,\uparrow}$	$15^{+5.6}_{-3.7}$
Intervalley scattering to upper spin split state	$\tau_{ph,\uparrow}$	$6^{+2.5}_{-1.9}$

Table S1: Fitting parameters with 90% confidence interval.

b- Model for low intensity and room temperature

Here, we now consider the Q valley populations. For simplicity, the spin-split states at the Q valleys have not been considered. The effect of a higher temperature is to add scattering channels to the Q valleys and to enhance intervalley scattering of the constituent holes. Here, we have added terms including intervalley hole scattering τ_{hole}, scattering to the nearest Q valley τ_{Q1} and scattering to the next nearest Q valley τ_{Q2}. Table S2 lists all the fitting parameters. All other additional scattering channels such as Q to Q valley scattering were found to be negligible.

Bright excitons:

$$\frac{dn_{K_\uparrow-K_\uparrow}}{dt} = g(t) - \left(\frac{1}{\tau_{ex}} + \frac{1}{\tau_{intra}} + \frac{1}{\tau_{ph}} + \frac{1}{\tau_{rad}} + \frac{1}{\tau_{hole}} + \frac{1}{\tau_{Q1}} + \frac{1}{\tau_{Q2}}\right) n_{K_\uparrow-K_\uparrow} + \frac{1}{\tau_{ex}} n_{K'_\downarrow-K'_\downarrow} + \frac{1}{\tau_{hole}} n_{K_\uparrow-K'_\downarrow} + \frac{1}{\tau_{intra,\uparrow}} n_{K_\downarrow-K_\uparrow} + \frac{1}{\tau_{ph,\uparrow}} n_{K'_\uparrow-K_\downarrow} \quad (11)$$

$$\frac{dn_{K'_\downarrow-K'_\downarrow}}{dt} = -\left(\frac{1}{\tau_{ex}} + \frac{1}{\tau_{intra}} + \frac{1}{\tau_{ph}} + \frac{1}{\tau_{rad}} + \frac{1}{\tau_{hole}} + \frac{1}{\tau_{Q1}} + \frac{1}{\tau_{Q2}}\right) n_{K'_\downarrow-K'_\downarrow} + \frac{1}{\tau_{ex}} n_{K_\uparrow-K_\uparrow} + \frac{1}{\tau_{hole}} n_{K'_\downarrow-K_\uparrow} + \frac{1}{\tau_{intra,\uparrow}} n_{K'_\uparrow-K'_\downarrow} + \frac{1}{\tau_{ph,\uparrow}} n_{K_\downarrow-K'_\downarrow} \quad (12)$$

Spin-dark excitons :

$$\frac{dn_{K_\downarrow-K_\uparrow}}{dt} = -\left(\frac{1}{\tau_{hole}} + \frac{1}{\tau_{intra,\uparrow}} + \frac{1}{\tau_{ph,\uparrow}}\right) n_{K_\downarrow-K_\uparrow} + \frac{1}{\tau_{intra}} n_{K_\uparrow-K_\uparrow} + \frac{1}{\tau_{ph}} n_{K'_\downarrow-K_\uparrow} + \frac{1}{\tau_{hole}} n_{K_\downarrow-K'_\downarrow} + \frac{1}{\tau_{Q1}} n_{Q-K_\uparrow} + \frac{1}{\tau_{Q2}} n_{Q'-K_\uparrow} \quad (13)$$

$$\frac{dn_{K'_\uparrow-K'_\downarrow}}{dt} = -\left(\frac{1}{\tau_{hole}} + \frac{1}{\tau_{intra,\uparrow}} + \frac{1}{\tau_{ph,\uparrow}}\right) n_{K'_\uparrow-K'_\downarrow} + \frac{1}{\tau_{intra}} n_{K'_\downarrow-K'_\downarrow} + \frac{1}{\tau_{ph}} n_{K_\uparrow-K'_\downarrow} + \frac{1}{\tau_{hole}} n_{K'_\uparrow-K_\uparrow} + \frac{1}{\tau_{Q1}} n_{Q'-K'_\downarrow} + \frac{1}{\tau_{Q2}} n_{Q-K'_\downarrow} \quad (14)$$

Spin-unlike momentum-dark excitons:

$$\frac{dn_{K_\uparrow-K'_\downarrow}}{dt}=-\left(\frac{1}{\tau_{intra}}+\frac{1}{\tau_{ph}}+\frac{1}{\tau_{hole}}+\frac{1}{\tau_{Q1}}+\frac{1}{\tau_{Q2}}\right)n_{K_\uparrow-K'_\downarrow}+\frac{1}{\tau_{hole}}n_{K_\uparrow-K_\uparrow}+\frac{1}{\tau_{intra,\uparrow}}n_{K_\downarrow-K'_\downarrow}$$

$$+\frac{1}{\tau_{ph,\uparrow}}n_{K'_\uparrow-K'_\downarrow} \tag{15}$$

$$\frac{dn_{K'_\downarrow-K_\uparrow}}{dt}=-\left(\frac{1}{\tau_{intra}}+\frac{1}{\tau_{ph}}+\frac{1}{\tau_{hole}}+\frac{1}{\tau_{Q1}}+\frac{1}{\tau_{Q2}}\right)n_{K'_\downarrow-K_\uparrow}+\frac{1}{\tau_{hole}}n_{K'_\downarrow-K'_\downarrow}+\frac{1}{\tau_{intra,\uparrow}}n_{K'_\uparrow-K_\uparrow}$$

$$+\frac{1}{\tau_{ph,\uparrow}}n_{K_\downarrow-K_\uparrow} \tag{16}$$

Spin-like momentum-dark excitons:

$$\frac{dn_{K_\downarrow-K'_\downarrow}}{dt}=-\left(\frac{1}{\tau_{hole}}+\frac{1}{\tau_{intra,\uparrow}}+\frac{1}{\tau_{ph,\uparrow}}\right)n_{K_\downarrow-K'_\downarrow}+\frac{1}{\tau_{intra}}n_{K_\uparrow-K'_\downarrow}+\frac{1}{\tau_{ph}}n_{K'_\downarrow-K'_\downarrow}+\frac{1}{\tau_{hole}}n_{K_\downarrow-K_\uparrow}$$

$$+\frac{1}{\tau_{Q1}}n_{Q-K'_\downarrow}+\frac{1}{\tau_{Q2}}n_{Q'-K'_\downarrow} \tag{17}$$

$$\frac{dn_{K'_\uparrow-K_\uparrow}}{dt}=-\left(\frac{1}{\tau_{hole}}+\frac{1}{\tau_{intra,\uparrow}}+\frac{1}{\tau_{ph,\uparrow}}\right)n_{K'_\uparrow-K_\uparrow}+\frac{1}{\tau_{intra}}n_{K'_\downarrow-K_\uparrow}+\frac{1}{\tau_{ph}}n_{K_\uparrow-K_\uparrow}+\frac{1}{\tau_{hole}}n_{K'_\uparrow-K'_\downarrow}$$

$$+\frac{1}{\tau_{Q1}}n_{Q'-K_\uparrow}+\frac{1}{\tau_{Q2}}n_{Q-K_\uparrow} \tag{18}$$

Q-valley momentum-dark excitons:

$$\frac{dn_{Q-K_\uparrow}}{dt}=-\left(\frac{1}{\tau_{hol}}+\frac{1}{\tau_{Q1}}+\frac{1}{\tau_{Q2}}\right)n_{Q-K_\uparrow}+\frac{1}{\tau_{hole}}n_{Q-K'_\downarrow}+\frac{1}{\tau_{Q1}}n_{K_\uparrow-K_\uparrow}+\frac{1}{\tau_{Q2}}n_{K'_\downarrow-K_\uparrow} \tag{19}$$

$$\frac{dn_{Q-K'_\downarrow}}{dt}=-\left(\frac{1}{\tau_{ho}}+\frac{1}{\tau_{Q1}}+\frac{1}{\tau_{Q2}}\right)n_{Q-K'_\downarrow}+\frac{1}{\tau_{hole}}n_{Q-K_\uparrow}+\frac{1}{\tau_{Q1}}n_{K_\uparrow-K'_\downarrow}+\frac{1}{\tau_{Q2}}n_{K'_\downarrow-K'_\downarrow} \tag{20}$$

$$\frac{dn_{Q'-K_\uparrow}}{dt}=-\left(\frac{1}{\tau_{hole}}+\frac{1}{\tau_{Q1}}+\frac{1}{\tau_{Q2}}\right)n_{Q'-K_\uparrow}+\frac{1}{\tau_{ho}}n_{Q'-K'_\downarrow}+\frac{1}{\tau_{Q1}}n_{K'_\downarrow-K_\uparrow}+\frac{1}{\tau_{Q2}}n_{K_\uparrow-K_\uparrow} \tag{21}$$

$$\frac{dn_{Q'-K'_\downarrow}}{dt}=-\left(\frac{1}{\tau_{hole}}+\frac{1}{\tau_{Q1}}+\frac{1}{\tau_{Q2}}\right)n_{Q'-K'_\downarrow}+\frac{1}{\tau_{hol}}n_{Q'-K_\uparrow}+\frac{1}{\tau_{Q1}}n_{K'_\downarrow-K'_\downarrow}+\frac{1}{\tau_{Q2}}n_{K_\uparrow-K'_\downarrow} \tag{22}$$

Fitted quantity	Fitting parameter	Value (ps)
Exchange scattering	τ_{ex}	$0.25^{+0.05}_{-0.04}$
Intravalley relaxation to lower spin split state	τ_{intra}	$1.7^{+1.1}_{-0.8}$
Intervalley phonon scattering	τ_{ph}	$0.16^{+0.02}_{-0.01}$
Radiative recombination	τ_{rad}	$0.25^{+0.01}_{-0.01}$
Intravalley scattering to upper spin split state	$\tau_{intra,\uparrow}$	11^{+6}_{-4}
Intervalley scattering to upper spin split state	$\tau_{ph,\uparrow}$	$0.34^{+0.13}_{-0.08}$
Scattering to nearest Q valley	τ_{Q1}	$0.5^{+0.08}_{-0.07}$
Scattering to next nearest Q valley	τ_{Q2}	$1.9^{+1}_{-0.7}$
Intervalley hole scattering	τ_{hole}	$1.1^{+0.4}_{-0.3}$

Table S2: Fitting parameters with 90% confidence interval.

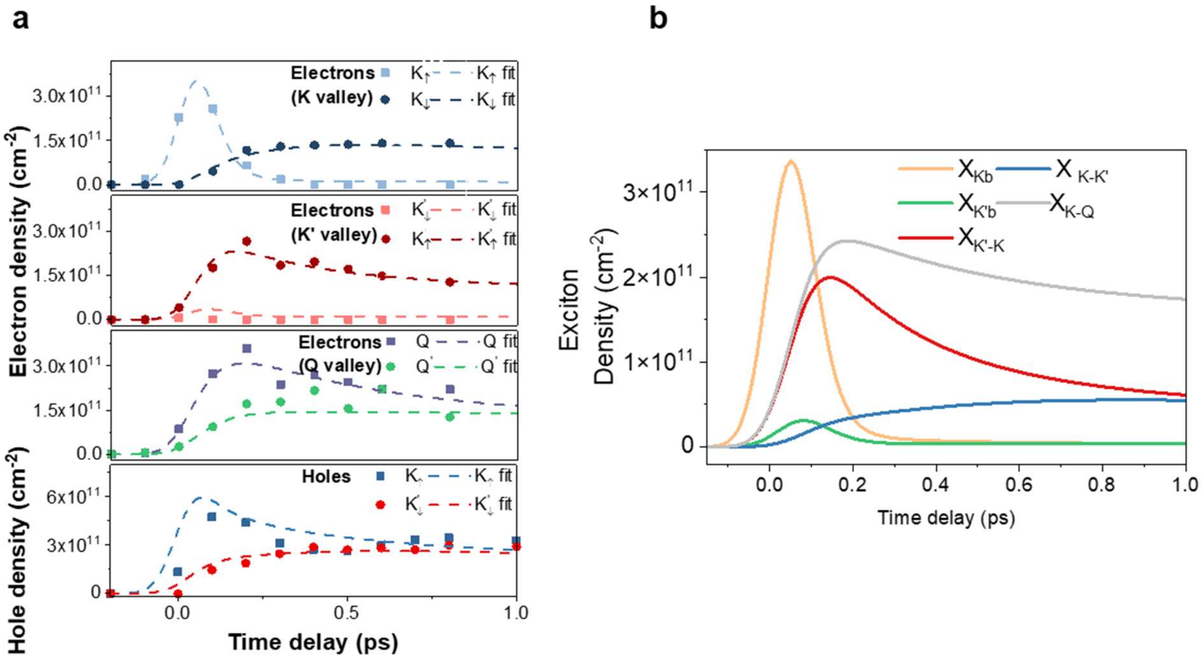

Fig.S8: Room temperature data and fit with rate equations. (a) Extracted electron and hole populations in the K and K' valleys (dots) and fittings from the room temperature model (dashed lines). (b) Dynamics of the bright excitons, momentum-dark excitons and K-Q excitons.

At room temperature, our model based on rate equations shows that the overall phonon-assisted scattering is enhanced. In particular, we see that the intervalley scattering becomes the main cause for the initial valley depolarization of the bright exciton by rapidly scattering to both K valley and Q valley momentum dark excitons. Furthermore, in contrast to the low temperature observations, now intervalley scattering of the hole is enabled which leads to the complete depolarization of dark states as well.

8. Spin dark excitons populations and degree of polarization

Using the rate equation described above we see that our data indicate that the spin dark states are the lowest energy states of the system. By plotting the ratio between the spin dark exciton and the momentum dark exciton populations for which the electron lies in the same valley, we see continuous increase of this ratio indicating that momentum dark excitons slowly relax to the spin dark states as previously observed in time-resolved photoluminescence experiments[5] .

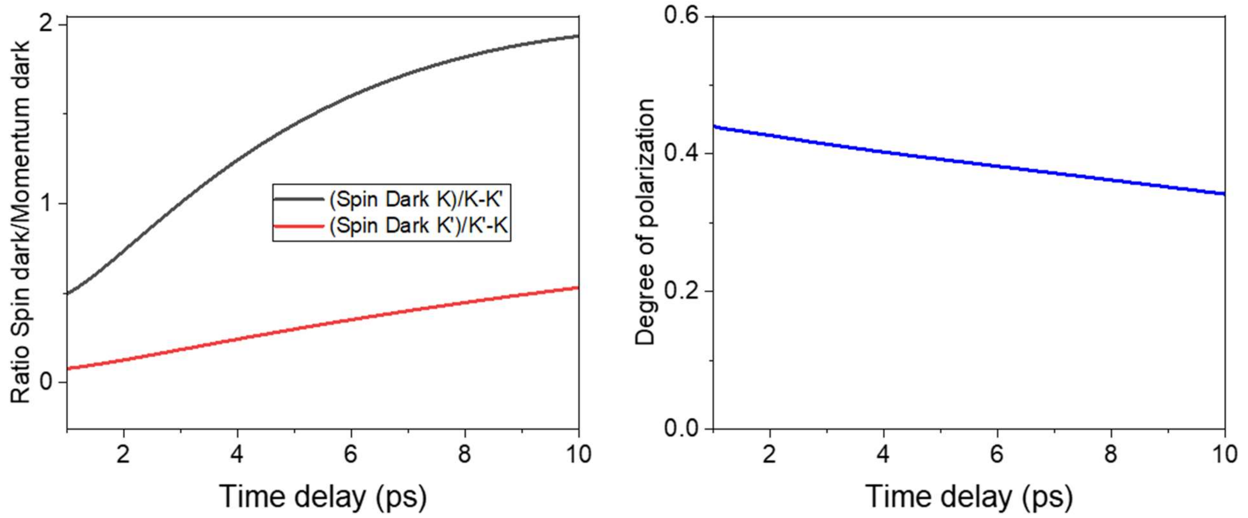

Fig.S9: Evolution of spin-dark and momentum Ratio between the spin dark K-valley exciton and the K-K' momentum dark exciton (black) and between the spin dark K'-valley exciton and K'-K momentum dark exciton (red). The degree of polarization for the spin dark K exciton is shown in blue.

9. Dispersion of the exciton electrons

To clarify that the observed negative dispersion of the electron signal in Fig. 2 is not a result of laser-assisted photoemission effects, we also extracted the dispersion at a later time delay of 5 ps. At this delay, the negative dispersion—characteristic of an excitonic state in ARPES—remains clearly visible in both the K and K' valleys (Fig. S10).

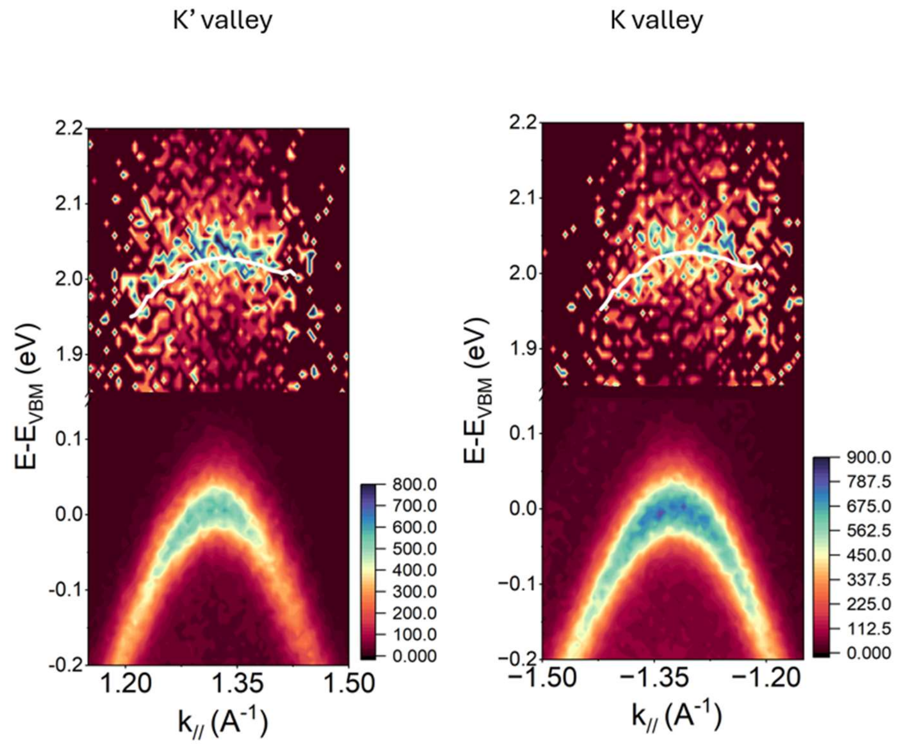

Fig.S10: Negative dispersion of the exciton-electron. Data at 5 ps time-delay showing the valence band and the exciton electron signals (normalized at each k vector). The dispersion was obtained by fitting with a Gaussian function the energy distribution at each k vector and plotting the peak of each Gaussian.

10. Intensity dependent momentum distribution of the bright exciton and valley polarization.

We have performed experiments to measure the K-valley bright exciton valley polarizationpolarization at 0.1 ps (corresponding to the maximum population of the K-valley bright exciton) for a range of density from 4×10^{11} cm^{-2} to 4×10^{12} cm^{-2}. First, we have extracted the momentum distribution of the electron signals (See Fig.S11), and, as density increases, we observe a broadening of the momentum distribution that is due to an increase of center-of-mass momentum as previously shown[6]. Then, we extracted the corresponding valley polarization of the K-valley bright exciton at the same time-delay, and we clearly see a decrease of valley polarization associated to the increase of the width of the momentum distribution, i.e. center-of-mass momentum, as one would expect from enhanced intervalley exchange interaction. This supports our interpretation that at low intensity, intervalley exchange interaction is suppressed.

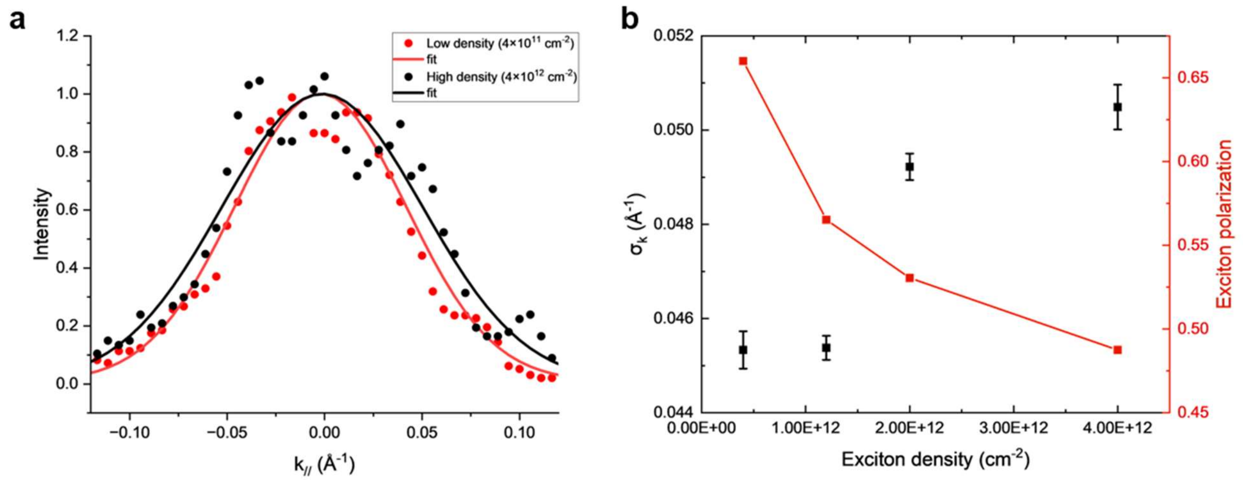

Fig.S11: Influence of intensity on the bright exciton momentum distribution and degree of valley polarization. (a) Momentum distribution at 0.1 ps of the electron signal from the bright exciton in the K valley at low and high intensity. (b) Momentum distribution width from Gaussian fitting with 90% confidence interval versus exciton density (black squares) and valley polarization of the K-valley bright exciton (red).

References

1. Shirley, E. L., Terminello, L. J., Santoni, A. & Himpsel, F. J. *Brillouin-Zone-Selection Effects in Graphite Photoelectron Angular Distributions*. *PHYSICAL REVIE%* vol. 8.

2. Madéo, J. *et al. Directly Visualizing the Momentum-Forbidden Dark Excitons and Their Dynamics in Atomically Thin Semiconductors*. https://www.science.org (2020).

3. Robert, C. *et al.* Exciton radiative lifetime in transition metal dichalcogenide monolayers. *Phys Rev B* **93**, (2016).

4. Koutenský, P. *et al.* Ultrafast Dynamics of Valley-Polarized Excitons in WSe2 Monolayer Studied by Few-Cycle Laser Pulses. *Nanomaterials* **13**, (2023).

5. Kusaba, S., Watanabe, K., Taniguchi, T., Yanagi, K. & Tanaka, K. Role of dark exciton states in the relaxation dynamics of bright 1s excitons in monolayer WSe2. *Appl Phys Lett* **119**, (2021).

6. Man, M. K. L.*et al. Experimental Measurement of the Intrinsic Excitonic Wave Function*. *Sci. Adv* vol. 7 https://www.science.org (2021).
